# A Multi-Scale and Multi-Level Fusion Approach for Deep Learning-Based Liver Lesion Diagnosis in Magnetic Resonance Images with Visual Explanation

**DOI:** 10.3390/life11060582

**Published:** 2021-06-18

**Authors:** Yuchai Wan, Zhongshu Zheng, Ran Liu, Zheng Zhu, Hongen Zhou, Xun Zhang, Said Boumaraf

**Affiliations:** 1 Beijing Key Laboratory of Big Data Technology for Food Safety, Beijing Technology and Business University, Beijing 100048, China; 2030702057@st.btbu.edu.cn (H.Z.); zhangxun@btbu.edu.cn (X.Z.); 2 Beijing Lab of Intelligent Information Technology, School of Computer Science, Beijing Institute of Technology, Beijing 100081, China; zhengzhongshu@bit.edu.cn; 3 China South-to-North Water Diversion Corporation Limited, Beijing 100038, China; liuran2011@163.com; 4 Department of Diagnostic Radiology, National Cancer Center/National Clinical Research Center for Cancer/Cancer Hospital, Chinese Academy of Medical Sciences and Peking Union Medical College, 17, Panjiayuan NanLi, Chaoyang District, Beijing 100021, China; 5 Centre d’Exploitation des Systèmes de Télécommunications Spatiales (CESTS), Agence Spatiale Algérienne, Algiers, Algeria; said.boumaraf@yahoo.com

**Keywords:** computer-aided diagnosis, liver cancer, deep learning, visual explanation, multi-scale representation, multi-level fusion

## Abstract

Many computer-aided diagnosis methods, especially ones with deep learning strategies, of liver cancers based on medical images have been proposed. However, most of such methods analyze the images under only one scale, and the deep learning models are always unexplainable. In this paper, we propose a deep learning-based multi-scale and multi-level fusing approach of CNNs for liver lesion diagnosis on magnetic resonance images, termed as MMF-CNN. We introduce a multi-scale representation strategy to encode both the local and semi-local complementary information of the images. To take advantage of the complementary information of multi-scale representations, we propose a multi-level fusion method to combine the information of both the feature level and the decision level hierarchically and generate a robust diagnostic classifier based on deep learning. We further explore the explanation of the diagnosis decision of the deep neural network through visualizing the areas of interest of the network. A new scoring method is designed to evaluate whether the attention maps can highlight the relevant radiological features. The explanation and visualization make the decision-making process of the deep neural network transparent for the clinicians. We apply our proposed approach to various state-of-the-art deep learning architectures. The experimental results demonstrate the effectiveness of our approach.

## 1. Introduction

Liver cancer, which mainly includes hepatocellular carcinoma in the setting of cirrhosis and cholangiocarcinoma, is a serious threat to human health [1]. The early detection and diagnosis of liver cancer is important to increase the chance of survival [2]. The biopsy is currently the golden standard for cancer diagnosis, but it is invasive and not always viable because of the location of the tumor [3,4]. Therefore, noninvasive imaging techniques, such as computed tomography (CT) and magnetic resonance imaging (MRI), are currently viewed as effective techniques for evaluating liver lesions. Indeed, based on the current worldwide recommendations, guidelines and guidance documents, it is even possible to make a diagnosis of hepatocellular carcinoma based on imaging criteria without the need for a biopsy [5,6,7,8,9,10]. However, imaging criteria are still limited in terms of sensitivity because of the need to maintain a high specificity for the diagnosis [5,9,10]. As such, many recent studies have been published thus far to improve the diagnosis of liver cancer, especially in the setting of cirrhosis, and even to predict the progression of a liver lesion into cancer [11,12,13,14]. Despite these improvements, many liver lesions, especially in the setting of cirrhosis, are still deemed indeterminate [9]. With the rapid development of artificial intelligence, the computer-aided diagnosis (CADx) technique on medical images gradually becomes one of the effective ways to automatically diagnose diseases early [15].

The CADx methods of liver lesions try to classify lesions into benign, malignant or different grades, automatically utilizing intelligent methods. In the traditional CADx frameworks, hand-crafted features are extracted as the representations of the lesions and then classified into different classes using traditional machine learning models, such as the support vector machine (SVM), adaboost and random forest (RF). With the development of deep learning techniques, deep learning-based CADx methods have arisen in recent years, especially the convolutional neural network (CNN) architecture. Deep learning methods brought obvious improvements to classification performance and became popular as time passed.

Although the deep learning-based liver lesion diagnosis methods work well, they still suffer from the following two problems: (1) most of such methods analyze the lesions in medical images under only one scale (such as 70*70 voxels in [16]), which makes it easy to ignore the overall feature information about lesions, resulting in unsatisfactory diagnosis performance, and (2) the CNN is an end-to-end model, and the details of the data flow inside it are invisible. Thus, it is hard to explain why and how the classification decision is made and whether the decision is trustworthy. There may exist cases where the CNN can gain perfect diagnosis performance, but the features used by the CNN to support the decisions are irrelevant or incorrect. Such cases are quite unacceptable in the medical imaging area, where a high degree of reliability is required. Therefore, it is essential to explain the diagnosis decisions of CNNs.

**Figure 1:**
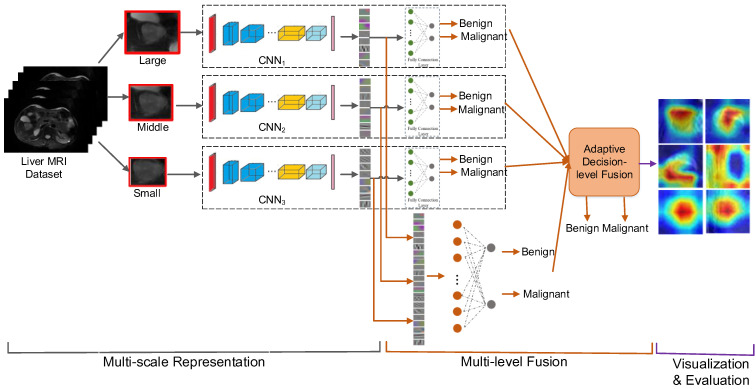
Framework of the proposed method for liver lesion diagnosis.

In this paper, we propose a deep learning method with multiscale and multi-level fusion of CNNs for liver lesion diagnosis in MR images, termed as MMF-CNN. Furthermore, we explore the interpretation of the diagnosis decision of the deep learning method. The framework of the proposed method is illustrated in Figure 1. To extract as much feature description of the lesions as possible, we explore large-, middle- and small-scale representations of liver lesions. Then, we construct CNNs to extract the discriminative features and classify the lesions into benign or malignant at different scales. As the features and classifiers at multiple scales may provide complementary information [17], we present a multi-level fusion method to combine them at the feature level and decision level hierarchically and arrive at a final diagnosis result. Different from the methods that terminate at the diagnosis results, we further work on the explanation of the decision-making process of the neural networks. We visualize the attention maps that show the areas of interest in the CNN and further design a new scoring method to evaluate whether the CNN can react to the true relevant radiological findings. With the explanation, the practitioner can make sure whether the diagnosis result of the CNN is trustworthy, and the deep learning-based CADx systems would build better trust and make a step forward in the clinical community.

In summary, the contributions of this paper are as follows:

(1) We introduce multi-scale representations of liver lesions. As the scales and shapes vary among different lesions in MR images, it is inadvisable to predefine the scale of patches in advance. The consideration of multi-scale representations can provide better feature description of the lesions.

(2) We propose the multi-level fusion method of CNNs to generate a more robust and stable classifier. At the feature level, we fuse the complementary feature information of multi-scale lesion patches using a deep neural network. Then, at the decision level, we propose an adaptive fusion method based on the Dempster–Shafer (D-S) evidence theory to fuse the classification decisions of multiple CNN classifiers. Through the multi-level hierarchical fusion, we mine the complementary information of multi-scale lesion patches and obtain the final diagnosis result.

(3) We propose a multiscale and multi-level CNN fusion framework which is applicative to any CNN structure, including the self-defined structure or the widely used typical structures. To show the superiority and generality of our method, we adopt it and compare it with different models in the experiments. Furthermore, we try to explain the difference in diagnostic performance among the different methods through projecting and visualizing the extracted features of images. The visualized features explained the performance difference.

(4) We explore the explanation of the diagnosis decision by providing attention maps that show the supporting areas of the decision. A new scoring method of attention maps is designed to evaluate whether the CNN classifiers can discover the true relevant radiological features. Through the explanation, we can provide the diagnosis decision and the reasons for making this decision simultaneously, which helps to improve the reliability of deep learning-based CADx methods.

(5) Our method provides a new possible pattern for CAD in the clinical field through providing the supports for diagnostic decision. Our visualized attention maps can promisingly act as an important reference. For a new scanned medical image, our deep learning diagnostic model can output the diagnostic decision and the attention map for supporting the decision simultaneously. The clinician can first observe the diagnosis result and then refer to the visualized attention map to make sure whether the diagnostic model focuses on the right regions and whether the diagnosis result of the deep learning model for this image is trustworthy.

The remainder of this paper is organized as follows. Section 2 describes the related works. Section 3 presents the details of our proposed MMF-CNN approach. The experimental results are discussed in Section 4. We conclude this work in Section 5.

## 2. Related Work

### 2.1. Traditional CADx Methods of Liver Lesions

The traditional diagnosis methods of liver lesions mainly consist of two stages: handcrafted feature extraction and lesion classification. The commonly used traditional handcrafted features for liver image analysis include gray level co-occurrence matrices (GLCM) [18,19], gray histograms [20], local binary patterns (LBPs) [21] and discrete wavelet transforms (DWTs) [18]. The widely used traditional classification models include the SVM [22,23], Bayes classifier [24], RF [25], artificial neural networks (ANNs) [26] and functional trees (FTs) [27]. By combining different feature extraction and classification models together, various diagnosis approaches can be generated. For example, Xian et al. [23] utilized the GLCM feature and SVM classifier to identify malignant and benign liver tumors from ultrasound images. Poonguzhali et al. [26] employed the GLCM feature and ANN classifier to detect texture differences in focal lesions and normal tissues in liver ultrasound images. Mougiakakou et al. [28] used different types of texture features and an ANN classification scheme for the classification of four types of hepatic tissues in CT images. For a detailed discussion of handcrafted features and classifiers, please refer to [27] and [29], in which reviews of different features and classifiers for computer-aided liver cancer diagnosis are presented.

The traditional CADx methods are easy to understand, explain and implement. Aside from that, the training of the models is efficient and does not need large-scale training data. However, the performance of the handcrafted features is highly correlated with the characteristics of the dataset. The handcrafted feature that is effective for one dataset may not work well for another dataset. What is more, the accuracy performance of the traditional CADx methods needs to be improved further.

### 2.2. Deep Learning-Based CADx Methods of Liver Lesions

In recent years, more and more attention has been attracted by deep learning-based CADx methods. Deep learning-based methods generally employ a deep neural network as the tool for target tasks, among which the CNN model is widely used. Generally, the CNN can be used in two manners. The first method utilizes a CNN as a feature extractor to extract innovative features from images. It allows the image itself to be used as the input of the neural network. Therefore, feature extraction methods based on a CNN enable much more information included in the extracted feature than the handcrafted methods. Shen et al. [30] used a CNN model to obtain a set of globally discriminative features in CT images. Then, the SVM and RF classifiers were employed to classify the extracted features as benign or malignant. Except for the feature extractor, the CNN is more commonly used as an end-to-end classifier system, employing the images as the input and outputting the classification results. Yasaka et al. [16] used a CNN for the classification of liver masses in dynamic contrast agent-enhanced CT. In the CNN, six convolutional layers, three maximum pooling layers and three fully connected layers were used. This method allowed for high discrimination between malignant versus category C and benign masses. However, the sensitivity for diagnosing category B masses was not good. Wu et al. [31] proposed a diagnostic system of liver disease classification based on contrast-enhanced ultrasound imaging, in which the deep belief network is employed to classify benign and malignant focal liver lesions. As is shown in this work, this method is better than the other compared methods such as linear discriminant analysis (LDA), K-nearest neighbors (KNN), the SVM and back propagation net (BPN) in terms of accuracy, sensitivity and specificity. Romero et al. [32] incorporated the feature extraction of inception V3 combined with residual connections for the discrimination between liver diseases in abdominal CT images. Compared with a texture-based approach trained with SVMs, Inception-ResNet-V2 and Inception-V3, the proposed model with pre-trained weights from ImageNet performed better in four out of the seven metrics, such as accuracy, balanced accuracy, F1 score and recall. Hassan et al. [33] used a CNN to calculate the latent features from unlabeled liver images in an unsupervised manner and then used a softmax layer to diagnose the different focal liver diseases. This proposed system achieved results of 97.2%, 98% and 95.7% in the criteria of accuracy, sensitivity and specificity, respectively, which were superior to the compared methods, such as the multi-support vector machine, KNN, and naive Bayes classifier. 

The deep learning-based CADx methods can extract more discriminative feature representations from the input images and gain high accuracy. However, the training of the deep learning model is time-consuming. Aside from that, the decisions of deep learning models are always hard to interpret.

### 2.3. Deep Neural Network Interpretation Methods

While the deep neural networks enable superior performance in multiple tasks, their lack of decomposability into intuitive and understandable components makes them hard to interpret [34]. Thus, deep neural networks are often referred to as black box models. Recently, researchers have made some efforts toward the explanation of deep neural networks. The corresponding interpretation methods can be generally divided into two strategies: closing the black boxes or opening the black boxes. The first strategy keeps the neural network as a black box and tries to design interpretable surrogate models to approximate the behavior of the neural network as closely as possible. For example, to explore which part of the original input image is important for the decision-making of the neural network, the authors of [35,36,37] segmented the original input image into different patches and then used traditional classifier models to classify the combination of different patch sets. Through comparison of the classification performances, each patch of the whole image is assigned with a value of importance. For the second strategy, it tries to open the black box and analyze the hidden layers of the neural network. Thus, the gradients, parameters and neurons in the neural network are analyzed, and heat maps [38,39,40] and activation map [41,42,43,44] are generated as the visualization results.

## 3. The Proposed Methods

Given a liver MR image, our goal is to output the classification result of benign or malignant. The framework of our MMF-CNN approach is illustrated in Figure 1, which consists of three parts: multiscale representation, multi-level fusion and visualization and evaluation. Details of these three parts are given in this section.

### 3.1. Multiscale Representation

**Figure 2:**
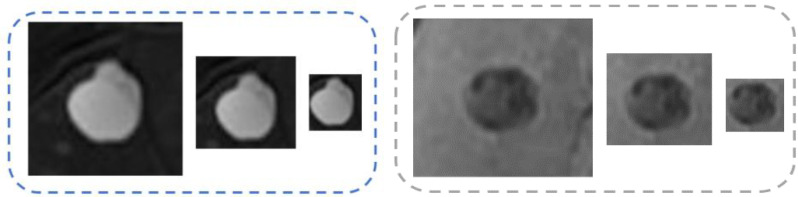
The examples of a benign lesion (left) and a malignant lesion (right), where the patches’ scales are 256 × 256, 128 × 128 and 64 × 64 in voxels.

We explore the patch-based method for diagnosis, where lesion patches cropped from the liver MR images are used as the input of the diagnosis framework. To extract both local and semi-local complementary feature information, we propose a multiscale sampling strategy. Three scales (large, middle and small) of patches were considered, such as 256 × 256, 128 × 128 and 64 × 64 in voxels, which were used in the experiments of our paper (Figure 2). For each lesion in the MR image, we cropped three scales of patches covering this lesion.

Due to the good performance of deep learning models, we used a CNN to learn the globally discriminative feature representations from the image patches. Three CNNs were built corresponding to the three scales, with each CNN trained using the patches of each respective scale. For the structure of the CNNs, our method is applicable for any structure, including the self-defined CNN structure or the widely used typical structures, such as Resnet, VGG or Alexnet in our experiments. For training these three CNNs, different strategies can be adopted, namely with or without transfer learning. Generally, for the case where the network contains large amount of parameters and have little training data, the transfer learning strategy can be used to support the training process. For the lightweight networks such as Enet [45], the network can be trained from the initial state, where the transfer learning is not adopted, like in the studies of Albert Comelli et al. [46] and Renato Cuocolo et al. [47]. In the experiments in our paper, we adopted the widely used large-scale networks and adopted the transfer learning strategy. Each CNN was first pre-trained using a traditional large set of images, such as the ImageNet database [48] used in the experiments of our paper, and then fine-tuned using the patches of the corresponding scales.

For each patch, we extracted the output of the feature extractor in the CNN to gain a feature vector as the representation of it. Thus, for a lesion in an MR image, three feature vectors were extracted corresponding to the three scales.

Aside from that, the last layer of each CNN output a binary diagnosis result about the patch of the corresponding scale. Therefore, each CNN was also used as a classifier.

### 3.2. Multi-Level Fusion

To integrate and take advantage of the multi-scale complementary feature information and increase the robustness of the classification, we fused the information at both the feature and decision levels.

#### 3.2.1. Feature-Level Fusion

Patches in different scales may contain different feature information of a lesion. Thus, we fused the three scales of patches at the feature level and designed a three-layer neural network for the fusion. We first concatenated the feature vectors of three scales of patches in the input layer. Then, after the computation in two fully connected layers, the diagnosis result of the fused feature was output.

For training this three-layer neural network for feature-level fusion, we initialized the parameters of the network randomly and used the liver MR images for parameter optimization.

#### 3.2.2. Decision-Level Fusion

Up until now, we have had four CNN classifiers built for classification of a liver lesion at the large, middle, small and feature-level fused scales. To obtain a more robust and stable classifier, we further fused the classification results of these four classifiers. An adaptive decision-level fusion method based on the D-S evidence theory is proposed for our fusion, which is described as follows.

**D-S Evidence Theory.** The D-S evidence theory [49] deals with the uncertain information reasoning problem. It considers each instance as a source of evidence and combines this evidence using Dempster’s rule. Let $\Theta =\left\{\theta_{1},\theta_{2},\ldots ,\theta_{n}\right\}$ be a finite set called the frame of discernment of a problem, where $\theta_{i}\;(i=1,2,\ldots ,n)$ are mutually exclusive and exhaustive elements. For each subset A of Θ, a probability can be assigned, which is called the basic probability assignment (BPA). The BPA represents the knowledge of the evidence about the problem, as well as the uncertainty incorporated in the information source. The definition of the BPA should satisfy the following constraints:

(1) $$\left\{ \begin{array}{l} m(\phi )=0\\ \sum_{A\subset \Theta }m(A)=1 \end{array}\right.$$

where ϕ is an empty set (i.e., the condition that cannot be true in any state).

Given w evidence over the same frame of discernment, this evidence can be combined into a common agreement using the following combination rule:

(2) $$\left\{ \begin{array}{l} m(A)=K^{-1}\sum_{\cap A_{i}=A}\prod_{i=1}^{w}m_{i}(A_{i})\\ K=\sum_{\cap A_{i}\neq \phi }\prod_{i=1}^{w}m_{i}(A_{i}) \end{array}\right.$$

In our work, the output of each of the four CNN classifiers can be regarded as an instance of evidence. The frame of discernment is Θ={b, m}, where b and m mean benign and malignant, respectively. A can be ϕ, {b}, {m} or {b,m}. A={b,m} reflects the uncertainty of the classifier for answering whether the classification is benign or malignant.

Then, we combined the classifier’s global classification performance for the whole dataset and the local performance on a certain lesion together to define the BPA. For each classifier i (i=1, 2, 3, 4), we designed the BPA for lesion j as

(3) $$\left\{ \begin{array}{l} m_{ij}(\phi )=0\\ m_{ij}(\left\{b\right\})=p_{b\_ij}*acc_{i}\\ m_{ij}(\left\{m\right\})=p_{m\_ij}*acc_{i}\\ m_{ij}(\left\{b,m\right\})=1-acc_{i} \end{array}\right.$$

where $acc_{i}$ is the classification accuracy of classifier i on the whole dataset and $m_{ij}(\left\{b,m\right\})=1-acc_{i}$ is the uncertainty of the classifier. $p_{b\_ij}$ and $p_{m\_ij}$ are the probabilities of being benign or malignant given by classifier i on lesion j. As the output values of the CNN classifiers were not in the range [0,1], we used a softmax function to transfer the outputs into probabilities to get $p_{b\_ij}$ and $p_{m\_ij}$.

**Our Adaptive Fusion Method.** In the fusion of the standard D-S evidence theory, there exists a problem: the answers given by different evidence may conflict. When this conflict happens, the fusion result may be mistaken, since it artificially increases the masses of the compromise hypotheses [50]. In order to deal with the situations with conflicts and without conflicts, we designed an adaptive fusion method. For a lesion, if no or low conflict exists in the four evidences, we adopt the standard D-S evidence theory method to fuse these four classifiers. Otherwise, we compute the credibility [51] of each evidence. Then, we select the evidence with the highest credibility and follow the decision of it as the fusion result. The credibility of one piece of evidence measures the degree that it is supported by other evidence, and high credibility means high importance among the evidence.

For the computation of credibility, we first compute the similarity matrix of these four examples evidence. For any two pieces of evidence $m_{i}$ and $m_{j}$ (i,j=1,2,3,4), we compute the similarity $s_{ij}$ between them by using the method in [50].

Then, the similarity matrix for the four classifiers is

(4) $$S=\left[\begin{array}{l} 1\;\;s_{12}\;s_{13}\;s_{14}\\ s_{21}\;1\;\;s_{23}\;s_{24}\\ s_{31}\;s_{32}\;1\;\;s_{34}\\ s_{41}\;s_{42}\;s_{43}\;1 \end{array}\right]$$

We add the values of line i and get the support degree $Sup(m_{i})$ for the evidence i:

(5) $$Sup(m_{i})=\sum_{k=1}^{4}s_{ik}$$

Then, the credibility is obtained by normalizing the support degrees using Equation (6):

(6) $$Crd(m_{i})=\frac{Sup(m_{i})}{\sum_{k=1}^{4}Sup(m_{k})}$$

Furthermore, the normalization coefficient K in Equation (2) is the conflict coefficient as well. A larger value for K means higher conflict exists among the evidence. Therefore, we used the value of K to determine whether the conflict existed.

Based on the conflict coefficient K and the computation of credibility, the adaptive fusion method was designed, and it is given in Algorithm 1.
**Algorithm 1.** The adaptive decision-level fusion method.**Inputs:** (1) the outputs of four CNN classifiers for one lesion      (2) the classification accuracy $acc_{i}$ of each classifier      (3) threshold T **Outputs:** the fused final classification decision**Steps:** 1. Transfer the outputs into probabilities using softmax function; 2. Compute the BPA of each classifier using Equation (3); 3. Compute the conflict coefficient K utilizing Equation (2); 4. If K is less than T: 5. fuse these four results using standard D-S evidence theory as shown in Equation (2) and get the final classification result of this lesion; 6. Else if K is greater than T: 7. compute the credibility of each CNN classifier using Equations (4)–(6); 8. select the classifier with the highest credibility, and follow the classification decision of it as the final classification result of this lesion. 

### 3.3. Visualization and Evaluation

The deep neural network is an end-to-end black box system. Therefore, we can obtain the classification result from a CNN, but it is hard to explain why and how the result is reached by the CNN and whether the diagnosis result is trustworthy. There may exist a case where the features utilized by the CNN for supporting the diagnosis decision are irrelevant to the certain disease. Thus, it is important to explore the explanation of the diagnosis process of the CNN. We designed the visualization step to show the supporting area of the diagnostic decision. Furthermore, to evaluate the visualization results, we designed a new scoring method to compare the performance of the CNNs.

**Figure 3:**
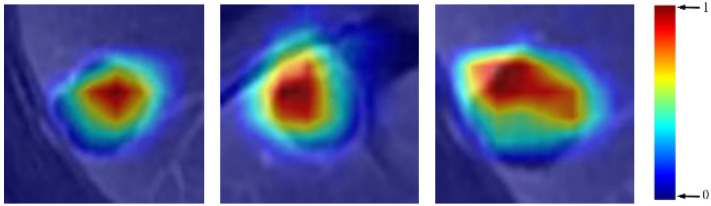
Examples of visualized attention maps.

**Visualization.** The last convolutional layer of a CNN contains rich semantic information and plays an important role in the decision-making process. Therefore, we analyzed the feature maps and gradients of the last convolutional layer of a CNN and generated a class-discriminative attention map using the gradient-weighted class activation maps (Grad-cam) method [39]. The attention map displays the areas of interest of the CNN in the decision-making process. The color bar was applied to the attention map to make a heat map and show the attention map more intuitively. We give examples of attention maps as heat maps in Figure 3, where the attention maps are overlaid onto the original lesion patch. Regions with different colors denote different degrees of importance for the decision-making of the CNN, where the red-colored regions have higher importance and the blue-colored regions have lower importance.

In our approach, we fused the CNNs corresponding to multiple scales. Thus, for a liver lesion, we could obtain different attention maps from different scales of CNNs. To select the optimal attention map, we computed the BPA of each CNN classifier using Equation 3 and selected the one with the highest BPA for visualization.

**Evaluation.** For the evaluation of the visualization results, most methods rely on subjective user ratings or voting [39,40,41], which makes it hard to give an objective comparison between the visualization results of deep neural networks. The attention maps reflect the areas of interest in CNNs. To evaluate whether the CNNs could react to true relevant radiological findings, we designed the scoring method to compare the visualized attention map of our method with the gold standard and calculate the consistency degree between them. In simulating the clinical diagnosis process of clinicians, we considered four sets of voxels about the lesion in a gold standard patch: (1) the voxels inside the lesion ($S_{1}$), (2) the edge voxels of the lesion ($S_{2}$), (3) the voxels outside the lesion but five voxels away from the edge ($S_{3}$) and (4) the other voxels in the image ($S_{4}$). As clinicians pay different levels of attention to these four types of voxels in the clinical diagnosis, we set different weights for these four sets of voxels. The weights corresponding to them are set as $w_{1}=1$, $w_{2}=0.5$, $w_{3}=0.2$ and $w_{4}=0$, as suggested by experienced clinicians. For the attention map from the CNN, we normalized the values in the attention map to the range [0,1] and computed the consistency Score between the attention maps from the CNN and the radiological finding:

(7) $$\left\{ \begin{array}{l} Score=\sum_{j=1}^{3}\sum_{i\in S_{j}}f(i)\;\\ f(i)=\left\{ \begin{array}{l} 1,\;if\;w_{j+1}\leq map_{i}\;\leq w_{j}\\ 0,\;otherwise \end{array}\right. \end{array}\right.$$

where $map_{i}$ denotes the attention map value for voxel i. Equaiton (7) means that for each voxel, if the value of the attention map fits in the scope of the corresponding region weights, the voxel is counted. The sum of the counted voxels is considered the consistency score. Accroding to Equation (7), a higher Score means that the areas of interest in the CNN fit the relevant radiological findings better, and vice versa.

## 4. Experiments and Results

### 4.1. Experimental Setup

**Dataset.** We evaluated the proposed method with the liver MRI dataset collected by the Cancer Institute and Hospital at the Chinese Academy of Medical Sciences. This dataset was obtained using a 3.0T scanner (GE Signa Excite HD), and it contains the MR images of 85 patients (35 benign with a cyst of the liver and 50 malignant with liver cancer). We conducted an axial plane T1WI, T2WI scan and a liver acquisition with volume acceleration (LAVA) dynamic multi-phase enhancement scan and obtained an average of 5 scans from each patient. In this way, we obtained a dataset of 425 images, containing 250 malignant and 175 benign scans. Among the 85 patients, the scans of 5 patients include more than one lesion. For each of these five patients, we randomly selected one lesion for the experiment. The lesions in the MR images were manually annotated by experienced radiologists to produce a gold standard.

**Compared Methods.** To test the robustness and the generality of our proposed approach, we applied it on various widely used state-of-the-art CNN architectures, including Resnet18 [52], VGG11 [53] and Alexnet [54]. For each CNN architecture, we compared the results of using our multiscale, multi-level fusing strategy and not using this fusing strategy (namely the commonly used single-scale framework in the image classification domain). For sufficient comparison, in the method without the fusing strategy, we utilized each of the scales (large, middle and small) for the experiments. These three CNN architectures were all pre-trained on the ImageNet [48] dataset and then fine-tuned for our dataset. To fit the input size of these CNN architectures, all patches were resized to the required size of the CNN architectures using bilinear interpolation.

Aside from that, we compared our method with the traditional classification methods. We considered the widely used handcrafted GLCM features of the lesions, with angles of 45°, 90° and 135° and distances of 1, 2 and 3. The contrast, dissimilarity, homogeneity, energy and correlation of the GLCM texture feature were computed. Three traditional classifiers, including AdaBoost, SVM and RF, were utilized to cooperate with the GLCM features to complete the classification task.

**Evaluation Criteria.** Five widely used measurements of classification performance were considered: the classification accuracy rate, sensitivity, specificity, positive predictive value (PPV) and negative predictive value (NPV). If a positive lesion was classified correctly by the algorithm, we called it ‘true positive’; otherwise, we called it ‘false negative’. The means of the ‘true negative’ and ‘false positive’ lesions were defined similarly. Let TP, TN, FP and FN be the number of true positives, true negatives, false positives and false negatives, respectively. Then, the accuracy, sensitivity, specificity, PPV and NPV are measured as in Equations (8)–(12), respectively:

(8) $$Accuracy=\frac{TP+TN}{TP+TN+FP+FN}$$

(9) $$Sensitivity=\frac{TP}{TP+FN}$$

(10) $$Specificity=\frac{TN}{TN+FP}$$

(11) $$PPV=\frac{TP}{TP+FP}$$

(12) $$NPV=\frac{TN}{FN+TN}$$

For the feature extraction ability, we projected the extracted features into 2D subspace using principal component analysis (PCA) and displayed the data in a 2D plane. Aside from that, we generated the attention maps to explain the decisions of the CNNs and scored them using Equation (8).

**Implementation Details.** We trained and evaluated our proposed approach and the compared methods using k-fold cross-validation. In our experiments, we set k as five by experience, like in many other similar studies [55,56]. The evaluation criteria were averaged to arrive at the final performance evaluation. We trained and optimized the CNN parameters using stochastic gradient descent (SGD) with momentum, with the learning rate, the batch size and the number of iterations set as 0.001, 10 and 500, respectively. The conflict threshold of K was set as 0.85. All the experiments were conducted on a computer with an Nvidia GeForce 1080 Ti GPU and 10 GB of memory.

### 4.2. Experimental Results

#### 4.2.1. Performance Comparisons

We conducted the liver lesion diagnosis task using our proposed approach on different CNN architectures and the compared classification methods and recorded the results of the accuracy rate, sensitivity, specificity, PPV and NPV. We present the results in groups, as shown in Table 1. In the group of each CNN architecture, we list the results of the commonly used single-scale strategy, our feature-level fusion and the multi-level fusion. 

From Table 1, we can conclude the following:

(1) The deep learning-based methods showed obviously superior performance to the traditional methods.

(2) Compared with the commonly used single-scale strategy, the feature-level fusion method could bring performance improvements in general. It achieved higher classification accuracy than the single-scale strategies for all of the three CNN architectures. As for the other four criteria, even though the feature-level fusion suffered a little decline in specificity and PPV on the Resnet18 architecture, it achieved obvious improvements in all of the four criteria for the VGG11 and Alexnet architectures.

(3) Our proposed multi-level fusion method obtained the best classification performance, which achieved the highest results in all of the five criteria on Resnet18, VGG11 and Alexnet.

(4) Through the comparisons in (2) and (3), the feature-level fusion achieved performance improvements compared with the single-scale strategy, and the multi-level fusion method brought further improvements on the basis of feature-level fusion. The comparisons demonstrated the effectiveness and necessity of both feature-level fusion and decision-level fusion in our MMF-CNN approach.

(5) Our multi-level fusion approach obtained preferable performance in all of the three tested state-of-the-art CNN architectures, which illustrates the good generality of our proposed approach.

#### 4.2.2. Explanation and Visualization

1. On Diagnostic Performance Difference

The image features used for classification are crucial for the diagnosis task. As the feature is the representation of an image, the quality of the features has great influence on the classification performance. To explain the difference in diagnostic performance among the different classification models, we visualized the features extracted from the lesions by different methods. For the deep features, we considered the concatenated feature vector of the multi-scale patches. For the traditional method, we considered the GLCM feature vector.

**Figure 4:**
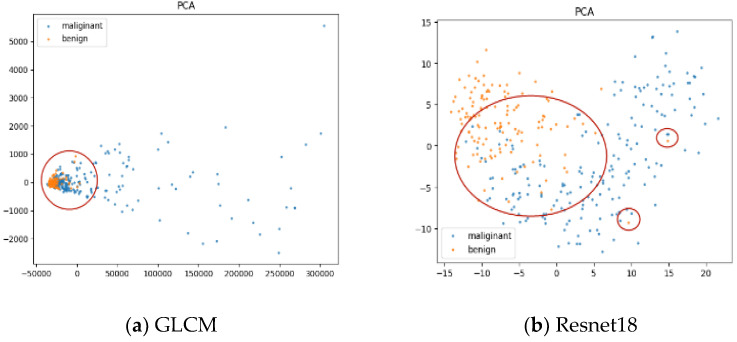

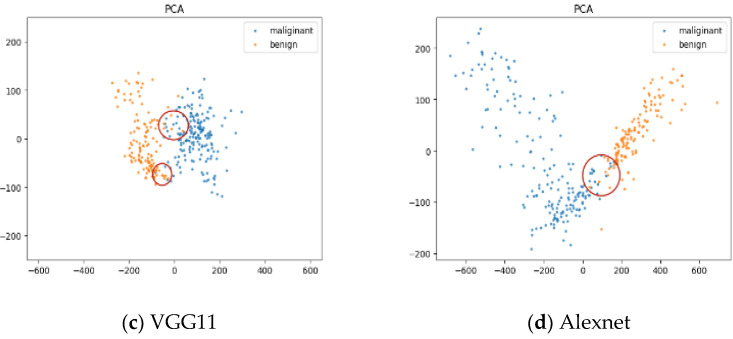
Visualization of feature vectors of different methods.

We projected the feature vectors into a 2D subspace using PCA and displayed the lesions in our dataset in a 2D plane. The results corresponding to the GLCM and each CNN architecture are shown in Figure 4, where the orange and blue markers denote benign and malignant lesions, respectively. In Figure 4, we circled the indistinguishable regions using red ellipses. We can see that the markers of the benign and malignant classes mixed heavily in Figure 4a. This shows that it was hard for the GLCM feature to divide the lesions into two classes clearly, while in Figure 4b–d, the markers are obviously discriminable in general, which demonstrates the good discriminatory ability of our concatenated multiscale features. From Figure 4b–d, the number of indistinguishable markers decreases gradually, which corresponds to the gradual improvements of the classification performance of Resnet18, VGG11 and Alexnet in Table 1. The visualized features explained the difference in diagnostic performance among the different methods.

2. On Decision-Making

**Figure 5:**
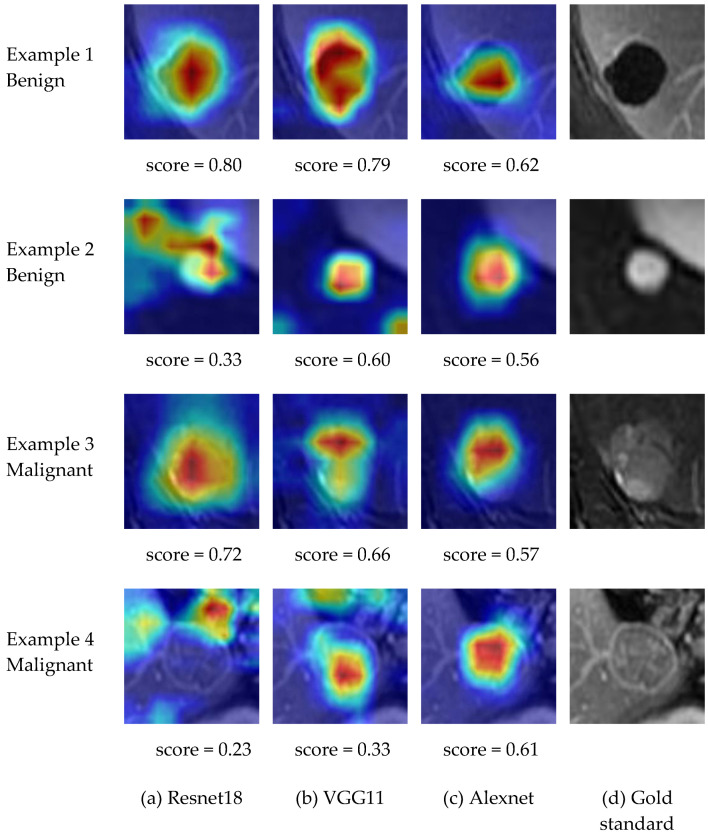
Examples of visualized attention maps of four lesions, with four columns corresponding to the results of Resnet18, VGG11, Alexnet and the gold standard.

For visual explanation of the classification decisions of the CNNs, we visualized the supporting areas in the decision-making process of the CNNs using the attention maps. We give examples of the visualized attention maps in Figure 5, with the Score given below each attention map. The four columns correspond to the results of Resnet18, VGG11, Alexnet and the gold standard. From Figure 5, we can see the following:

(1) In most cases, the attention maps could highlight the true relevant radiological findings (the gold standard) automatically.

(2) The Scores reflected the performance of the CNN architectures consistently. If the interests of the CNN focused on the lesion features or the attention map covered the lesion consistently, the score would be high, such as in the first example for Resnet18 and VGG11. If the interest area of the CNN architecture missed the lesion region, the score would be low, such as in the fourth example for Resnet18. Furthermore, we computed the average Score for all the lesions in the dataset for each CNN architecture. The results are shown in Table 2. For Resnet18, even though it identified the lesion region features better for some examples (shown in Figure 5), it failed in some other lesion examples. The instability of Resnet18 among the whole dataset led to the relatively low classification performance in Table 1 and relatively low score in Table 2. Alexnet showed a better average identification performance with the dataset; therefore, it achieved the best classification performance and attention map score. The consistency between the classification performance and the average Score of a CNN architecture demonstrates the effectiveness of our designed scoring method of attention maps.

The explanation of the decision-making process of the CNN classifier allows the CADx methods to make a step forward toward clinical applications. In the clinical applications of our approach, for a lesion in the MR image, our MMF-CNN approach can provide the diagnosis result and the attention map of the CNN classifier simultaneously to the medical practitioner. Referring to the attention map, the practitioner can make sure that the diagnosis result of the CNN for this lesion is trustworthy. Through the interaction, the clinical liver lesion diagnosis can benefit from the high efficiency of CADx systems and obtain high diagnosis accuracy and reliability at the same time.

## 5. Conclusions

In this paper, an explainable deep learning method with a multiscale and multi-level fusion of CNNs, termed as MMF-CNN, has been proposed for discriminating liver lesions into benign or malignant in MR images. We considered three scales of patches for each lesion to extract complementary feature descriptions and designed the multi-level fusion approach for the diagnosis of lesions. Both feature-level fusion and decision-level fusion were considered for integrating the complementary information of three scales of patches to gain a robust and stable classifier. We explained the method from two aspects, namely the explanation of the diagnostic performance difference and the explanation of the decision-making process. The visualized features explained the difference in diagnostic performance. The visualized attention map provided support for the decision-making process. We applied our approach to different state-of-the-art CNN architectures. The experimental results showed that the feature-level fusion achieved performance improvements compared with the single-scale strategy, and the multi-level fusion method brought further improvements on the basis of feature-level fusion. The comparisons demonstrated the effectiveness and necessity of both feature-level fusion and decision-level fusion in our MMF-CNN approach.

However, our work has the following limits: (1) the scale of the dataset used for the experiments was small, so the methods need to be compared in a larger dataset; (2) the interpretation of the deep learning model was quite simple and not enough, so more explanation about the diagnosis model needed to be made.

In the future work, we will conduct the comparison of different learning strategies of the training process, such as with or without transfer learning. Aside from that, we will collect the MRI scans of more patients and conduct more external validation. We will also introduce the attention mechanism into the diagnostic model to improve the diagnostic accuracy further. Aside from that, with the aim of making the liver diagnostic model more transparent to users, we will explore more explanation and visualization methods of deep learning models, such as visualizing the hidden layers, the neurons and the gradients.

## Figures and Tables

**Table 1:** Performance comparison for liver lesion classification of different methods.

Method		Accuracy (%)	Sensitivity (%)	Specificity (%)	PPV (%)	NPV (%)
Traditional	GLCM + AdaBoost	85.31	87.97	81.23	87.31	75.45
	GLCM + SVM	84.30	93.20	71.62	82.53	75.64
	GLCM + RF	87.09	90.69	81.63	88.02	80.83
Resnet18	Large Scale	92.20	94.34	88.63	92.45	91.64
	Middle Scale	90.58	92.77	87.38	91.52	89.17
	Small Scale	92.15	92.94	91.00	93.83	89.79
	Feature-Level Fusion	92.56	94.47	89.75	93.17	91.65
	Multi-Level Fusion	95.70	97.02	93.75	95.80	95.54
VGG11	Large Scale	94.02	95.74	91.50	94.32	93.67
	Middle Scale	95.65	96.94	93.75	95.80	95.43
	Small Scale	94.28	94.89	93.38	95.48	92.59
	Feature-Level Fusion	97.97	98.30	97.50	98.30	97.53
	Multi-Level Fusion	98.99	98.72	99.38	99.57	98.15
Alexnet	Large Scale	96.60	97.45	95.38	96.91	96.15
	Middle Scale	95.04	95.74	94.00	95.91	93.77
	Small Scale	98.03	98.21	97.75	98.51	97.39
	Feature-Level Fusion	98.86	98.93	98.75	99.14	98.46
	Multi-Level Fusion	99.49	99.15	100	100	98.77

**Table 2:** The average scores of the attention maps for different CNN architectures.

	Resnet18	VGG11	Alexnet
Average Score	0.45	0. 51	0.52
